# Public Knowledge, Attitude, and Practice Toward Vitamin D Deficiency in Al-Qunfudhah Governorate, Saudi Arabia

**DOI:** 10.7759/cureus.33756

**Published:** 2023-01-13

**Authors:** Safa H Alkalash, Mosad Odah, Haneen H Alkenani, Nouf H Hibili, Reem S Al-essa, Razan T Almowallad, Safiah Aldabali

**Affiliations:** 1 Community Medicine and Health Care, Umm Al-Qura University, Al-Qunfudhah, SAU; 2 Family Medicine, Menoufiya University, Shebin Alkom, EGY; 3 Biochemistry, Umm Al-Qura University, Al-Qunfudhah, SAU; 4 Medicine, Umm Al-Qura University, Al-Qunfudhah, SAU; 5 Medicine, Al-Qunfudhah College of Medicine, Umm Al-Qura University, Al-Qunfudhah, SAU

**Keywords:** vitamin d deficiency, vitamin d, practice, knowledge, attitude, awareness

## Abstract

Background: Vitamin D is a very important component of all vital functions in the human body. Its deficiency is a major public health issue worldwide and is associated with a broad spectrum of diseases. This study assessed knowledge, attitude, and practices regarding vitamin D deficiency among the general population in the Al-Qunfudhah governorate, Saudi Arabia.

Methods: An analytical cross-sectional study was carried out among the population in Al-Qunfudhah governorate, Saudi Arabia. A self-administrated online questionnaire was utilized to collect the research data during a period of four months from November 2021 to February 2022.

Results: A sample of 466 participants was recruited in this study, about two-thirds of them were females (64.4%) and had a university education (67.8%). Despite 91% of them having previously heard about vitamin D, only (17.4%) were able to recognize sunlight exposure as a main source of vitamin D. Poor knowledge and positive attitude regarding vitamin D were obviously seen among 72.3% and 95.7%. Although 89% of the participants' family members had been diagnosed with hypovitaminosis D. Only 45% of the sample were willing to be compliant with vitamin D supplement whenever it is needed. The most reported source of information regarding vitamin D among the respondents was mass media (62.2%). The associated variables of good knowledge were female gender (*P* 0.001), young (*P* 0.001), unmarried (*P *0.006), highly educated (*P* 0.048), and receiving medical information from physicians (*P *0.018).

Conclusion: This study reveals the poor level of knowledge about vitamin D deficiency among the Al-Qunfudhah population, and this negatively affected their compliance for vitamin D supplementation when having hypovitaminosis D. Positive attitude toward vitamin D deficiency among the majority of the participants was obvious and may direct them to change their behavior toward vitamin D. Therefore, this study highlights the necessity of educating and sensitizing population about vitamin D and prevention of its deficiency.

## Introduction

Vitamin D is an essential fat-soluble vitamin that is produced in the skin depending on sunlight and found in different food elements including oily fish, egg yolks, veal, beef, liver, sun-dried mushrooms, offal, milk, and dairy products, as well as nutritional supplements [1]. Since its discovery, the role of vitamin D has gotten extreme attention, from being a simple vitamin to a steroid prohormone. The most established purpose of vitamin D is to help calcium absorption from the gastrointestinal tract, hence ensuring adequate serum calcium and phosphate levels in the blood [2]. It is important in a variety of vital processes, including mineralization and skeleton growth, control of the parathyroid gland's function, and the immune system as a whole. It helps to prevent autoimmune disorders, chronic diseases, and malignancies, additionally, it supports the cardiovascular system's health [3,4].

The main circulating form of vitamin D is 25-hydroxyvitamin D (25(OH) D). A serum 25(OH) D concentration of less than 25 nmol/L is considered vitamin D deficiency and less than 50 nmol/L reflects inadequacy [5]. On the other side, exogenous hypervitaminosis D (>150 ng/mL) is usually due to the intake of vitamin D supplements in higher doses (excess of 10,000 IU/day) for a prolonged duration (months), which is more than the recommended upper limit (4,000 IU/day. However, this toxicity does not apply with abnormally high exposure of skin to the sun or by eating a diversified diet [6,7].

Vitamin D deficiency can be attributed to many different factors, including insufficient vitamin D in the diet, malabsorption, and poor usage also increased demands, increased excretion, and catabolism. Besides that, there are numerous conditions that influence vitamin D bioavailability, such as gastrointestinal problems that limit absorption; The problems of the kidneys and liver can inhibit the activation of parenteral vitamin D or affect its conversion to active metabolites [8]. Vitamin D deficiency is correlated to rheumatoid arthritis and other autoimmune diseases such as multiple sclerosis. There is a substantial link between vitamin D deficiency and the chance of suffering chronic diseases. Vitamin D levels below 20 ng/mL have been linked to a 30%-50% greater risk of colon, prostate, and breast cancers, as well as a higher mortality rate from these malignancies, according to another research. Lately, vitamin D deficiency has been connected to the severity of COVID-19-related symptoms [2].

The prevalence of vitamin D deficiency in the Middle East and North Africa ranged between 12%-96% in children and adolescents, and 54%-90% in pregnant women. In adults, it ranged between 44% and 96% [9]. In the Kingdom of Saudi Arabia, the overall vitamin D deficiency is 63.5% [10]. Saudi women have a significantly higher prevalence of VDD (41.2%-100%) than Saudi men (32.5%-92.6%). Additionally, vitamin D deficiency in infants (88%-90%), children, and teenagers (40.6%-97.8%) [8]. Vitamin D deficiency in the Saudi Arabian population is mainly due to limited exposure to sunlight, and subsequently, inadequate skin synthesis [1]. This occurs as a result of numerous factors, the climate desert of Saudi Arabia is characterized by extreme heat during the daytime and can be unaffordable, most people spend more of their time indoors in air-conditioned spaces and urban lifestyle, conservative clothing style, economic and social situation, dark skin pigmentation, which affects endogenous vitamin D production in addition to a lack of awareness [1,5].

Vitamin D deficiency is the most frequent dietary deficiency and one of the world's most common misdiagnosed medical problems [11]. Accordingly, raising public awareness about the importance of vitamin D can assist in significantly minimize health-related issues caused by its deficiency. Therefore, the current study's aim was to assess vitamin D public knowledge, as well as attitudes and practice toward vitamin D deficiency.

## Materials and methods

Study design and setting

This analytical cross-sectional study examined the knowledge of vitamin D deficiency along with attitudes and activities among the Al-Qunfudhah general population. The whole study duration (starting from selecting the study topic till reaching to publication process) was a 16-month period from August 2021 to December 2022. A self-administrated online survey was utilized to collect the research data.

The study setting was Al-Qunfudhah, which is a Saudi governorate located in the Tihamah plain on the Red Sea coast. It is located in Makkah Province. Its area is estimated at 5,195 km², which occupies about 3.7% of the regional area. It is considered a terminal area as it is far away from Jeddah and Mecca Almokarama (about 400 km), which are the nearest two big cities. Al-Qunfudhah governorate has a desert climate with its location close to the equator, making summers difficult to define, as a sequence its population cannot expose to the sun directly.

Study sample

The sample size was calculated by using EPI InfoTM (CDC, Atlanta, GA) [12] based on the total population number in the Al-Qunfudhah governorate of 300,516 and the frequency of good knowledge of 50%. At CI (95%), and a 5% margin of error. Therefore, the calculated sample size was 384 participants.

Procedure and tool for data collection

The required data were collected in a time frame of four months, from November 2021 to February 2022, through an online survey designed using Google Docs and distributed virtually on different electronic applications such as WhatsApp, Telegram, and Twitter.

The questionnaire was designed by the study researchers after a literature review then the relevant information was added, and its items were generated and drafted in the Arabic language as a 34-item questionnaire. The items were organized within the questionnaire with the help of an expert panel consisting of three members (Biochemistry, Family Medicine, and Internal Medicine) followed by pre-testing through the application of a pilot study.

Pilot study

Forty-three responses were analyzed; the survey was slightly amended prior to its use, to reflect the outcomes of the pilot trial. All data from this pilot study were excluded from the main study results as its role was to guide the study investigators and based on its results, some modifications were done to the designed survey like simplifying some of its items to be understandable by the public. Finally, questionnaire reliability was confirmed through the test-retest technique. Internal consistency was estimated to recognize the level at which the questionnaire's items measure the required data and the level to which their items are connected to each other. Cronbach's Alpha coefficient test was (0.80).

The applied survey included 34 assessment questions which were categorized into four sections. The first section included items about the participants' demographics (age, gender, marital status, educational level, and employment). The second section assessed the general awareness regarding vitamin D and the clinical manifestations of its deficiency. The third part studied their attitude about vitamin D deficiency, and the final section assessed the subjects’ practices with regard to vitamin D.

Scoring of knowledge and attitude

The knowledge score ranged from 0 to 26, with a mean knowledge score of 14.1 ± 3.9 out of 26 points. The value of knowledge, which was lower than the mean scores, was considered poor knowledge while the value at and above the mean scores was considered good knowledge. Regarding attitude score, a participant who correctly answered three or more questions out of four is considered as having a positive attitude.

The total number of collected questionnaires was 510. Incomplete and invalid questionnaires (n = 44) were discarded. The final valid, and complete questionnaires were 466.

Ethical considerations: Ethical approval was provided by the Medical Research and Ethical Committee of the college of medicine in Makkah with IRB of UQU reference (UCOJ060521). The applied questionnaire started with an opening question to obtain consent from all participants. Data confidentiality was maintained all the time.

Statistical analysis

After data were extracted, it was revised, encoded, and fed to SPSS version 22 (IBM, Inc., Chicago, IL). All statistical analysis was done using two-tailed tests. P-value less than 0.05 was statistically significant. Descriptive analysis based on frequency and percent distribution was done for all variables, including participants’ personal data, and education level, and occupation. Also, participants' knowledge, attitude, and practice regarding vitamin D and its deficiency were tabulated and overall awareness was graphed. Cross tabulation was used to assess factors affecting participants' knowledge and attitude toward vitamin D and deficiency. Pearson chi-squared test was applied to identify relations between dependent and independent variables and the exact probability test for small frequency distributions.

## Results

A total of 466 participants took part in the administered electronic survey. Most of the study subjects were female (n=300, 64.4%). Most of them were university educated (n=316, 67.8%). Considering marital status, (n=278, 59.7%) were not married (Table 1).

**Table 1:** Socio-demographic data of the study population. All values presented as numbers (N.) and percentages (%). #: Others= Intermediate/Primary education/Illiterate

Socio-demographic data	No	%
Gender		
Male	166	35.6%
Female	300	64.4%
Age in years		
< 18	26	5.6%
18-24	192	41.2%
25-35	149	32.0%
> 35	99	21.2%
Marital status		
Unmarried	278	59.7%
Married	188	40.3%
Education		
Bachelor’s degree	316	67.8%
Diploma	34	7.3%
High school	97	20.8%
Others^#^	19	4.1%
Occupation		
Employed	159	34.1%
Freelancer	9	1.9%
Student	183	39.3%
Retired	20	4.3%
Unemployed	95	20.4%

Around 91% of the study participants were aware of vitamin D and 88% knew that vitamin D is present in the body. As for the sources of vitamin D, 17.4% knew about sunlight exposure. Regarding the benefits of vitamin D, they reported; it strengthens bones (80.3%) and regulates the level of calcium in the body (57.5%). Exact 51.7% confirmed their awareness as regards causes of vitamin D deficiency (Table 2).

**Table 2:** Public knowledge regarding vitamin D deficiency. All values presented as numbers (N.) and percentages (%).

Knowledge items		No	%
Previously heard about Vitamin D	Yes	424	91.0%
	No	42	9.0%
Vitamin D present in the body	Yes	410	88.0%
	No	30	6.4%
	Don't know	26	5.6%
Sources of Vitamin D	Sunlight Exposure	81	17.4%
	Food supplements (multivitamins)	9	1.9%
	Certain Foods	2	0.4%
	All of the above	362	77.7%
	Don't know	12	2.6%
Health related benefits of vitamin D	Strengthens bones	374	80.3%
	Regulates the level of calcium in the body	268	57.5%
	Relieves muscle pain	238	51.1%
	Reduces cardiovascular disease	138	29.6%
	Irritable bowel syndrome treatment	41	8.8%
	Prevent bleeding	35	7.1%
	Stomach cancer treatment	33	7.5%
	Don’t know	48	10.3%
Causes of vitamin D deficiency	Yes	241	51.7%
	No	108	23.2%
	Maybe	117	25.1%
There is a relation between Vitamin D and Calcium levels	Yes	411	88.2%
	No	55	11.8%
Vitamin D deficiency is correlated with Osteoporosis	Yes	434	93.1%
	No	32	6.9%
Vitamin D improves immunity	Yes	335	71.9%
	No	20	4.3%
	Maybe	111	23.8%
Vitamin D deficiency linked with depression	Yes	315	67.6%
	No	40	8.6%
	Maybe	111	23.8%
Meals alone can provide sufficient levels of Vitamin D	Yes	149	32.0%
	No	161	34.5%
	Maybe	156	33.5%
Aware of normal serum Vitamin D levels	Yes	180	38.6%
	No	286	61.4%
Complications vitamin D deficiency	Osteoporosis	390	89.4%
	Hair loss	328	75.2%
	Depression	310	71.1%
	Acne	130	29.8%
	Obesity	75	17.2%
	Cancer	55	12.6%
	Eczema	45	10.3%
	Peptic ulcer	30	6.9%
	Blindness	18	4.1%
Overall score	0-26		
Mean ± SD	14.1 ± 3.9		

**Figure 1:**
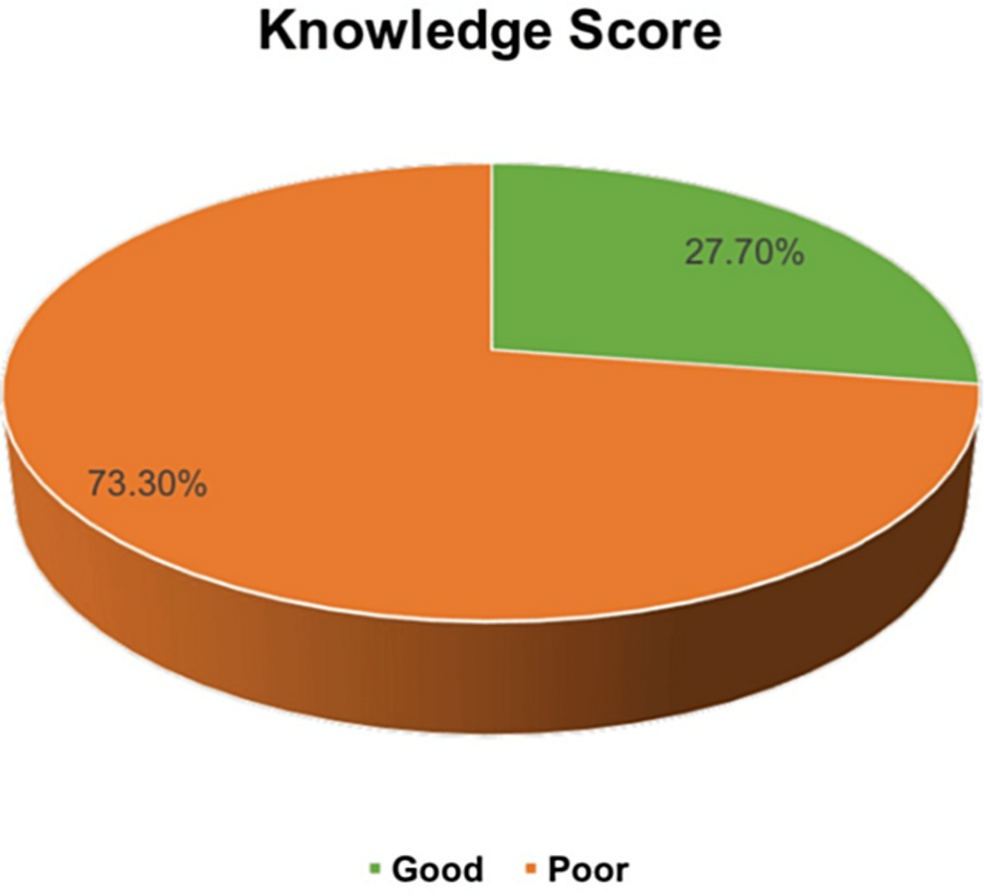
Public knowledge score regarding vitamin D deficiency.

A total of 337 (72.3%) participants had a poor knowledge level regarding vitamin D and its deficiency (Figure 1).

**Figure 2:**
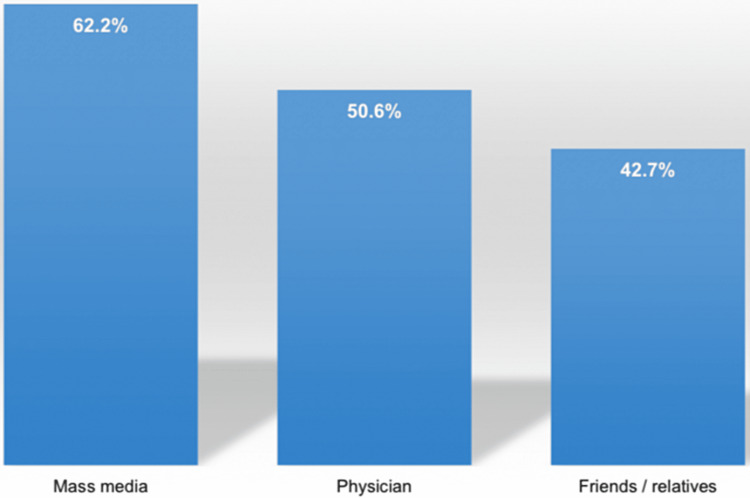
Sources of knowledge regarding vitamin D deficiency.

The most reported source information regarding vitamin D deficiency among the respondents was mass media (62.2%) (Figure 2). 

Exactly 99.6% of the study participants agreed that vitamin D is vital for overall health, 96.1% were interested to know about vitamin D deficiency-associated symptoms, 89.3% were concerned about vitamin D deficiency, and 75.3% like to get exposed to sunlight (Table 3).

**Table 3:** Public attitude toward vitamin D deficiency. All values presented as numbers (N.) and percentages (%).

Attitude items	No	%
Vitamin D is vital for overall health		
Yes	464	99.6%
No	2	0.4%
Exposure to sunlight		
Yes	351	75.3%
No	115	24.7%
Concerned about Vitamin D deficiency		
Yes	416	89.3%
No	50	10.7%
Interested to know about Vitamin D deficiency associated symptoms		
Yes	448	96.1%
No	18	3.9%

**Figure 3:**
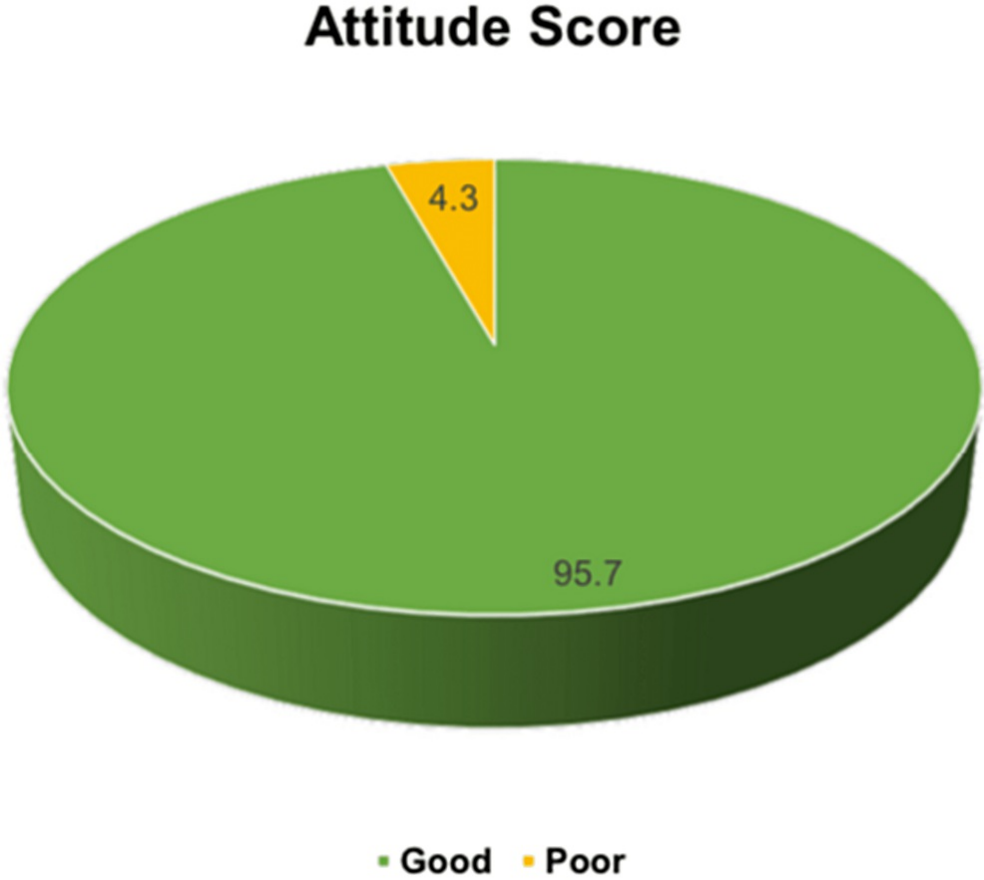
Attitude score of the participants toward vitamin D deficiency

The majority (95.7%) of the participants had positive attitude toward vitamin D (Figure 3).

Exactly 45.1% of the study participants self-reported doing vitamin D serum level tests while 74.2% reported assessing vitamin D serum levels in their family that were vitamin D deficient among 89.0% of them. A total of 57.7% reported using food supplements and 55.4% were prescribed vitamin D with a specific dosage. About 45.0% of them were willing to take vitamin D regularly. As for sun exposure, only 41.4% were exposed for 15-30 minutes daily (Table 4). 

**Table 4:** Public practice as regards vitamin D. All values presented as numbers (N.) and percentages (%).

Practice items	No	%
Previously investigated Vitamin D serum level test		
Yes	210	45.1%
No	256	54.9%
Taking food supplements		
Yes	269	57.7%
No	197	42.3%
Previously investigated Vitamin D serum levels among family members		
Yes	346	74.2%
No	120	25.8%
If yes, what was the result?		
Normal Vitamin D levels	35	10.1%
Vitamin D deficiency	308	89.0%
Excess Vitamin D levels	3	0.9%
Physician prescribed Vitamin D with specific dosage		
Yes	258	55.4%
No	208	44.6%
Willing to take Vitamin D regularly		
Yes	209	44.8%
No	257	55.2%
Get exposed to sunlight regularly		
Yes	203	43.6%
No	263	56.4%
Duration of daily sun exposure for whom expose to sunlight(n=203)		
Less than 15 minutes everyday	94	46.3%
15-30 minutes everyday	84	41.4%
More than 30 minutes everyday	25	12.3%
Prioritize eating vitamin D rich foods		
Yes	340	73.0%
No	126	27.0%

Variables affecting knowledge of participants toward vitamin D deficiency: Good knowledge was detected among 33.3% of females, 38.5% whose age group was 18-24 years, 32.4% of unmarried and highly educated participants (P-values 0.001, 0.001, 0.006, 0.048, respectively) (Table 5).

**Table 5:** Variables associated with good knowledge of participants toward vitamin D deficiency. All values presented as numbers (N.) and percentages (%). P: Pearson X2 test, $: Exact probability test, * P < 0.05 (significant) Others #: Intermediate/Primary education/Illiterate

Factors	Good knowledge level		P value
	No	%	
Gender			0.001*
Male	29	17.5%	
Female	100	33.3%	
Age group (in years)			0.001*
< 18	7	26.9%	
18-24	74	38.5%	
25-35	27	18.1%	
> 35	21	21.2%	
Marital status			0.006*
Unmarried	90	32.4%	
Married	39	20.7%	
Education			0.048*
Bachelor’s degree	96	30.4%	
Diploma	4	11.8%	
High school	22	22.7%	
Others^#^	7	36.8%	
Occupation			0.001*
Employed	30	18.9%	
Freelancer	1	11.1%	
Student	72	39.3%	
Retired	2	10.0%	
Unemployed	24	25.3%	
Source of information			0.001*
Physician	87	36.9%	
Mass media	87	30.0%	
Friends / relatives	53	26.6%	

Variables affecting the attitude of participants toward vitamin D deficiency: Considering positive attitude, it was significantly higher among married participants (98.4%; P=0.018). Furthermore, 98.7% of those who had received their information from physicians had a positive attitude toward vitamin D (P=0.001) (Table 6).

**Table 6:** Variables associated with positive attitude of participants towards vitamin D deficiency. All values presented as numbers (N.) and percentages (%). P: Pearson X2 test, $: Exact probability test, * P < 0.05 (significant) Intermediate/Primary education/Illiterate :#

Factors	Postive attitude level		P-value
	No	%	
Gender			0.935
Male	159	95.8%	
Female	287	95.7%	
Age group (in years)			0.846^$^
< 18	25	96.2%	
18-24	182	94.8%	
25-35	143	96.0%	
> 35	96	97.0%	
Marital status			0.018*
Unmarried	261	93.9%	
Married	185	98.4%	
Education			0.746^$^
Bachelor’s degree	304	96.2%	
Diploma	33	97.1%	
High school	91	93.8%	
#Others	18	94.7%	
Occupation			0.589^$^
Employed	154	96.9%	
Freelancer	8	88.9%	
Student	174	95.1%	
Retired	20	100.0%	
Unemployed	90	94.7%	
Source of information			0.001*
Physician	233	98.7%	
Mass media	281	96.9%	
Friends / relatives	185	93.0%	

## Discussion

Vitamin D deficiency is one of the most common preventive disorders that has a worldwide prevalence. Thus, it is critical to understand the public’s level of awareness and attitudes toward it and to recognize gaps in daily practices that would negatively affect vitamin D levels in their bodies. The current analytical cross-sectional community-based study assessed levels of knowledge, attitude, and practice about vitamin D deficiency among the Al-Qunfudhah population. The study found that the majority of its subjects had previously heard about vitamin D and knew its benefits to the human body, and the health-related issues in case of a decrease in vitamin D levels in the body. Although they recognized vitamin D sources, they did not know that the sun is its main source. These findings are in harmony with other studies [1,13]. The root cause of the similarity may be due to the analogy in the characteristics of its participants where the majority of them were females.

The studied sample denied their awareness of the etiology of vitamin D deficiency and could not identify whether diet alone is sufficient for getting adequate vitamin D or not. This gap in knowledge was previously denoted by Shaheen et al. and Alshamsan et al., in their studies where they found poor awareness levels about different exogenous sources of vitamin D [13,14]. This gap can be corrected by conducting awareness lectures for the public focusing on different causes of vitamin D deficiency and the important sources to maintain adequate body levels of vitamin D.

Poor knowledge score was detected among the majority of this study subjects, but this was an unexpected finding given that these days, there are many different means to know and understand health-related issues. This result is in concurrence with two Egyptian studies done on mothers in the Delta region, both studies reported the poor knowledge level among Egyptian mothers regarding vitamin D and its deficiency [13, 15]. The congruity in the results could be related to the similarity in culture and background in the two neighboring countries. While Conner et al., in their population-based study in England, found that more than half of their study sample was having a good level of knowledge about vitamin D [16]. This finding is better than the recent findings. The discrepancy between both outcomes may be caused by the dissimilarities in the cultural aspects and health care systems - between the two study settings.

Unfortunately, this study detected that social media is the main source of information among the studied population group, and this could explain the poor quality of knowledge. This is not the only study that found social media as a main source of population knowledge about vitamin D, a Jordanian study extracted the same outcome [17]. Furthermore, this outcome guided the current study’s researchers to inquire about the accuracy of health-related data, which is introduced by non-professionals on social media. Additionally, policies should be elaborated to filter and ensure consistency of the data that crosses world boundaries. The effect of social media on public health is a critical issue and should be investigated well to find the most suitable and applicable solutions to limit its role in health-related issues.

In spite of the poor knowledge level about vitamin D among the currently studied Saudi population, the majority (95.7%) of them possessed a positive attitude toward it. This is the same as the findings established by Uzrail et al. [17] in Jordan, where more than half of the study sample had a positive attitude toward vitamin D and the importance of sun exposure for adequate time. The explanation for this positive attitude may be due to the indirect message which was obtained by the participants after submitting their answers to the survey questions as regards vitamin D, its health-related benefits, and complications of its deficiency.

In this study, about half of the respondents had already investigated their serum levels of vitamin D. The majority (78%) of their families assessed it as well but unfortunately, a large proportion of them suffered from vitamin D deficiency. This was an expected outcome because many previous studies in Saudi Arabia [4,10,18] concluded the higher prevalence of vitamin D deficiency among its population and explained the main causes as the higher degree of temperature that withstands the correct and healthy sunlight exposure, besides the culture and religious aspects where women and girls should cover all her bodies even faces. Only about a third of the studied group expose their skin to sunlight for 15-30 minutes daily, which is similar to a study in Egypt [13]. This low percentage of persons who were keen to get sufficient sunlight exposure is critical because it increases the risk of vitamin D deficiency among them.

Being a young adult, highly educated, unmarried, female, and getting information from the physicians - all were associated with adequate knowledge about vitamin D. Previous studies in Saudi Arabia, Egypt, and Jordan support the recent study outcomes [2,13,17]. The rationale is that young, educated individuals are accustomed to using the internet to search for information, additionally, they had freshly graduated after taking studies in schools and universities. A positive attitude was associated with being married and receiving information from the physicians. Married individuals are enthusiastic to provide care for their families. Physicians can encourage people to take care of their health and any health-related issues.

There were some limitations of this study that should be acknowledged. The cross-sectional study design limits the causality of the findings. The online survey is another limitation that might carry a risk for non-response bias and may lead to different characteristics between the non-respondents and the respondents as there are many individuals in the community who are unable to submit the online survey thus their knowledge, attitude, and practice was ignored. However, the study investigators tried to maximize the sample size by forwarding the survey link via many social media platforms, and by expanding the duration of data collection in order to subdue the influence of these biases. However, this study is an initiative to spotlight an important health-related issue like vitamin D deficiency and identify the determinants for its occurrence and provide recommendations to change it.

## Conclusions

The current study detected a poor level of knowledge about vitamin D deficiency among the Al-Qunfudhah population, and in turn, adversely affected their adherence to vitamin D supplementation on having hypovitaminosis D. On the flip side, they possessed a positive attitude toward vitamin D and this may help them to change their behavior toward vitamin D. Therefore, health professionals and public health authorities should be encouraged to place further emphasis on combating vitamin D deficiency. Potential approaches include food fortification for the general population, and vitamin D supplementation in vulnerable individuals such as children, pregnant women, and the elderly. Another approach is advocating lifestyle modifications such as outdoor physical activity for the avoidance of obesity with careful and balanced sunlight exposure and a healthy diet. Finally, routine and regular serum levels of vitamin D should be investigated among members of the public, especially vulnerable groups such as children and the elderly as well as pregnant and lactating women.
